# Integrated Analysis of Global mRNA and Protein Expression Data in HEK293 Cells Overexpressing PRL-1

**DOI:** 10.1371/journal.pone.0072977

**Published:** 2013-09-03

**Authors:** Carmen M. Dumaual, Boyd A. Steere, Chad D. Walls, Mu Wang, Zhong-Yin Zhang, Stephen K. Randall

**Affiliations:** 1 Department of Biology, Indiana University-Purdue University Indianapolis, Indianapolis, Indiana, United States of America; 2 Lilly Research Laboratories, Eli Lilly and Company, Indianapolis, Indiana, United States of America; 3 Department of Biochemistry and Molecular Biology, Indiana University School of Medicine, Indianapolis, Indiana, United States of America; Institute of molecular and cell biology, Singapore

## Abstract

**Background:**

The protein tyrosine phosphatase PRL-1 represents a putative oncogene with wide-ranging cellular effects. Overexpression of PRL-1 can promote cell proliferation, survival, migration, invasion, and metastasis, but the underlying mechanisms by which it influences these processes remain poorly understood.

**Methodology:**

To increase our comprehension of PRL-1 mediated signaling events, we employed transcriptional profiling (DNA microarray) and proteomics (mass spectrometry) to perform a thorough characterization of the global molecular changes in gene expression that occur in response to stable PRL-1 overexpression in a relevant model system (HEK293).

**Principal Findings:**

Overexpression of PRL-1 led to several significant changes in the mRNA and protein expression profiles of HEK293 cells. The differentially expressed gene set was highly enriched in genes involved in cytoskeletal remodeling, integrin-mediated cell-matrix adhesion, and RNA recognition and splicing. In particular, members of the Rho signaling pathway and molecules that converge on this pathway were heavily influenced by PRL-1 overexpression, supporting observations from previous studies that link PRL-1 to the Rho GTPase signaling network. In addition, several genes not previously associated with PRL-1 were found to be significantly altered by its expression. Most notable among these were Filamin A, RhoGDIα, SPARC, hnRNPH2, and PRDX2.

**Conclusions and Significance:**

This systems-level approach sheds new light on the molecular networks underlying PRL-1 action and presents several novel directions for future, hypothesis-based studies.

## Introduction

The PRL family of enzymes has recently emerged as potential tumor biomarkers and novel anti-cancer therapeutic targets. Evidence suggests that the three PRL family members (PRL-1, PRL-2, and PRL-3) may be multi-faceted molecules involved in a number of diverse biological processes [1]–[5] owever recent attention to these enzymes revolves around their relationship to cellular proliferation and tumor progression.

PRL-1, the first family member identified, was initially characterized and named Phosphatase of Regenerating Liver for its role as an immediate early gene induced in mitogen-stimulated cells and in proliferating rat liver during hepatic regeneration [6], [7]. Accumulating evidence now indicates that up-regulation of PRL-1 expression can play a causal role in cellular transformation and tumor advancement. Overexpression of PRL-1 in non-tumorigenic cells leads to rapid cellular growth and a transformed phenotype [6], [8], [9]. Moreover, cells that stably overexpress PRL-1 exhibit enhanced cell motility and invasive activity and are capable of forming metastatic tumors in nude mice [6], [10]–[3]. Conversely, knockdown of endogenous PRL-1 in tumor cells has the opposite effect, reducing proliferation and suppressing cell migration and invasion [10], [12], [14]–[6]. An association between PRL-1 expression and tumor promotion has also been found in human tumor tissues where we previously showed that PRL-1 was significantly up-regulated in 100% of hepatocellular and gastric carcinomas compared to matched normal tissues from the same patients [17]. Collectively, these results suggest that the PRL-1 phosphatase regulates key pathways involved in tumorigenesis and metastasis. However, nearly 20 years now after its initial discovery, the mechanisms of PRL-1 action and regulation are still poorly understood and the exact biological function of this molecule remains unknown.

The focused study of individual, pre-selected molecules and pathways reveals that PRL-1 may be involved in multiple different signaling cascades. PRL-1 interacts directly with several phosphoinositide lipids [16], the cytoskeletal component α-tubulin [18], the Rho GTPase activating protein (GAP) p115 RhoGAP [19], the suppressor of TNF-mediated apoptosis TNFAIP8 (tumor necrosis factor alpha-induced protein 8) [20], the pro-survival transcription factor ATF-7 [21], and with FKBP38 (peptidylprolyl cis/trans isomerase FK506-binding protein 38), whose binding may target PRL-1 for proteosomal degradation [22]. PRL-1 over- or underexpression has been tied to alterations in expression of cell cycle regulators, such as Cyclin A, Cdk2, p21^cip1/waf1^, and p53 [9], [23]; focal adhesion complex proteins like FAK, Src, p130Cas, and paxillin [11], [12], [14]; the Rho GTPases RhoA, Rac1, and Cdc42 [10], [12], [14]; and the MAPK/ERK1/2 signaling cascade [11]. Additionally, PRL-1 is subject to redox regulation and has been suggested to play a role in the photo-oxidative stress response in the retina, where it relies on the glutathione system for constant regeneration of its enzymatic activity [5], [24].

It is clear from the variety of molecules it is capable of influencing or interacting with that PRL-1 signaling is multi-dimensional. However, no studies have yet examined the influence of PRL-1 expression on a broad scale. Therefore, the aim of the current study was to globally examine the gene and protein level alterations that occur downstream of PRL-1 in human embryonic kidney 293 (HEK293) cells, which are known to undergo cellular transformation and acquisition of a migratory, invasive, and metastatic phenotype in response to PRL-1 overexpression [11], [16]. Since “Omics” techniques offer the advantage of unbiased global analysis coupled to the opportunity to identify previously unforeseen players in a signaling network, we utilized microarray profiling of gene expression and mass spectrometry to broadly examine the influence of PRL-1 overexpression on the HEK293 transcriptome and proteome. This integrated, systems-level approach provides an unprecedented, comprehensive dataset that helps shed light on the molecular networks underlying PRL-1 action and identifies several potential downstream targets which can be examined in future, hypothesis driven studies.

## Methods

### Stable Cell Lines and Cell Culture

Human embryonic kidney 293 cells stably overexpressing PRL-1 (HEK293-PRL-1) or empty pcDNA4 vector (HEK293-vector) were previously generated and described [11], [16]. Cells were grown in 100 mm plates in Dulbecco's Modified Eagle's Medium (DMEM) supplemented with 10% (v/v) fetal bovine serum (Thermo Scientific HyClone, Logan, UT), 50 units/ml penicillin (Mediatech, Inc., Manassas, VA), and 50 µg/ml streptomycin (Mediatech).

### Mass Spectrometry

Seven 100 mm plates each of HEK293-PRL-1 and HEK293-vector cells were grown to 95% confluency, the culture medium was aspirated and the cell monolayers were washed once in 1X PBS, then frozen at −80C until use. Upon thawing the cells, protein samples were prepared and analyzed as previously described [25]. Briefly, cells were treated with 100 µl of a hypotonic lysis buffer containing 8M urea, 10 mM DTT and 1 mM sodium orthovanadate (Sigma-Aldrich, St. Louis, MO). The resulting protein lysates were reduced by triethylphosphine (Sigma-Aldrich), alkylated by iodoethanol (Sigma-Aldrich) and subsequently digested using Trypsin Gold (Promega, Madison, WI). Peptide concentration was determined using the Bradford Protein Assay.

Mass spectrometry (MS) and MS data analysis were carried out at Monarch Life Sciences (Indianapolis, IN) using previously described methods [25]–[28]. Tryptic digests were analyzed using a linear ion-trap mass spectrometer (LTQ) coupled to a Surveyor HPLC system (Thermo Scientific, Waltham, MA). Using a randomized schedule, tryptic peptides were injected (∼20µg/injection) onto a microbore, C18 reversed-phase column (Zorbax 300SB0-C18, 1 mm x 5 cm) with a flow rate of 50µl/min and eluted with a gradient from 5 to 45% acetonitrile (Honeywell Burdick & Jackson, Morristown, NJ) developed over 120 min. The effluent was electrosprayed into the LTQ mass spectrometer and data were collected in triple-play mode (MS scan, zoom scan, MS/MS scan). The acquired data were filtered and analyzed using a proprietary algorithm developed and described by Higgs et al. [26]–[28]. For peptide identification, database searches were carried out against the IPI (International Protein Index) human database and the non-redundant Homo sapiens database (NCBI) using both the X!Tandem and SEQUEST algorithms [29], [30]. Identified proteins were categorized into four tier groups (1–4) based on the quality of the peptide identification and the number of unique peptides identified. Proteins assigned to Tier 1 had high (>90%) peptide identification confidence and multiple sequences identified; Tier 2 had high peptide identification confidence, but with only a single peptide sequence identified; Tier 3 had moderate (75–89%) peptide identification confidence and multiple sequences; Tier 4 had moderate peptide identification confidence and a single sequence. Estimation of confidence levels was based on a random forest recursive partition supervised learning algorithm [26]. Only peptides assigned to proteins with a confidence level of greater than 90% (Tier 1 and Tier 2 peptides) were used for figures in this study, and only Tier 1 results were used for quantitative analyses. For protein quantification, raw files were acquired from the LTQ mass spectrometer and all extracted chromatograms (XIC) were aligned by retention time. To be used in the protein quantification procedure, each aligned peak must match parent ion, charge state, fragment ions (MS/MS data) and retention time (within a one-minute window). After alignment, the area-under-the-curve (AUC) for each individually aligned peak from each sample was quantile normalized, measured, and compared for relative abundance. All peak intensities were transformed to a log_2_ scale before quantile normalization. If multiple peptides had the same protein identification, then their quantile normalized log_2_ intensities were averaged to obtain log_2_ protein intensities. Analysis of Variance (ANOVA) was used to detect significant changes in protein expression between the HEK293-PRL-1 and HEK293-vector groups. The q-value threshold was fixed to control the false discovery rate (FDR) at 5% (≤0.05). The inverse log_2_ of each sample mean was calculated to determine the fold change between samples.

IPI identifiers and NCBI (National Center for Biotechnology Information) GenInfo Identifiers (GIs) were mapped to NCBI gene symbols using Biobase (http://www.biobase-international.com/) and the NCBI databases (http://www.ncbi.nlm.nih.gov/gquery). This mapping of proteins to their coding genes serves as the basis for integrating the protein mass-spectrometry results with the mRNA data sets described below.

### Gene Expression Microarray

Total RNA was extracted from three independent cultures each of HEK293-PRL-1 and HEK293-vector cells using the TRIzol reagent (Invitrogen Life Technologies, Carlsbad, CA) and further purified with an RNeasy Mini Kit (Qiagen Inc., Valencia, CA), following manufacturer's instructions. RNA integrity and yield were assessed by determining sample absorbance at 260 and 280 nm on a DU640B spectrophotometer (Beckman Coulter, Brea, CA) and by subjecting samples to the Agilent 2100 Bioanalyzer (Agilent Technologies, Inc., Santa Clara, CA), using the Agilent RNA 6000 Nano LabChip Kit as directed.

Gene expression profiling was carried out according to the protocol described in the Affymetrix GeneChip Expression Analysis Technical Manual. Briefly, 5µg of each cleaned, total RNA was used to generate double-stranded cDNA, by reverse transcription, using a Superscript Double-Stranded cDNA Synthesis Kit (Invitrogen Life Technologies) and a GeneChip T7-Oligo(dT) Promoter Primer Kit (Affymetrix, Santa Clara, CA). Following second-strand synthesis, cDNA was cleaned with a GeneChip Sample Cleanup Module (Affymetrix), and then used as a template for synthesis of biotinylated cRNA with the Enzo BioArray HighYield RNA Transcript Labeling Kit (Enzo Life Sciences, Farmingdale, NY). Labeled cRNA was cleaned with a GeneChip Sample Cleanup Module (Affymetrix), fragmented, and hybridized overnight to HG-U133 Plus 2.0 GeneChip Human Genome Arrays (Affymetrix), which analyze the expression level of more than 47,000 RNA transcripts and variants. Following hybridization, GeneChips were washed, stained with streptavidin phycoerythrin (Molecular Probes, a subsidiary of Life Technologies, Carlsbad, CA), and scanned using the Affymetrix GeneChip Scanner 3000 7G. Raw image (CEL file) generation and analysis was performed using the Affymetrix GeneChip Operating System (GCOS). All RNA samples and arrays met Affymetrix recommendations for standard quality control metrics.

Microarray data files were processed with R-project software (http://www.r-project.org/), version 2.13.1 through the RStudio interface version 0.94.92 (http://www.rstudio.org). The intensity values were read using the “affy” library of the Bioconductor package, version 2.8 [31], [32]. Normalization and calls were made using the Microarray Analysis Suite 5.0 (MAS5) procedure under default parameters. Probesets were scored for hybridization reliability as “High”, “Medium”, or “Low” by the method described in [33]. One of the chips that was hybridized with a HEK293-PRL-1 sample was removed from the analysis, after quantitative real-time reverse transcription PCR (qRT-PCR) confirmation revealed that this sample did not express PRL-1 differently from the controls, leaving 2 biological replicates in the PRL-1 overexpressing group and 3 biological replicates in the vector control group. Probesets that were not called as ‘present’ by MAS5 in at least four out of the five remaining chips were removed from the analysis, save for cases where a probeset was present in both members of the PRL-1 overexpressing group but absent in all of the vector controls. 15,967 probesets of the original 54,675 passed this presence filter. The loss of one expression chip from the data set increased the significantly detectable fold-change in a 2-way unpaired ANOVA test by 35%, thus resulting in our observing fewer significant mRNA expression changes than we potentially could have detected otherwise.

After transformation into a log_2_ scale, mean normalized expression values were calculated for each of the 15,967 probesets over all biological replicates for both of the experimental comparison groups (HEK293-PRL-1 and HEK293-vector). Differential expression between the two groups was determined for each probeset and assessed for significance in terms of p*-*value by the Student's t*-*test. Multiple-testing FDR correction values were calculated using the Benjamini-Hochberg procedure [34].

### Quantitative RT-PCR

A set of 184 genes, identified by microarray and/or proteomic analyses as differentially regulated or associated in the literature with signaling pathways involved in integrin-mediated cell signaling, cytoskeletal remodeling, and/or cell motility, was chosen for validation of gene expression changes using qRT-PCR. Total RNA was isolated as described for the microarray experiments, but using independent biological replicates of HEK293-PRL-1 and HEK293-vector cells. Isolated total RNA was treated with DNase I, using the Ambion TURBO DNA-free kit from Invitrogen Life Technologies and 1µg of each sample was reverse transcribed into cDNA with the SuperScript III First-Strand Synthesis System and random hexamer primers (Invitrogen Life Technologies), in accordance with the manufacturer's guidelines. The resulting cDNA was used as template for qRT-PCR using commercially available TaqMan Gene Expression Assays (Applied Biosystems, a subsidiary of Life Technologies, Carlsbad, CA) custom arrayed on 96-well plates. Table S2 contains the full list of TaqMan assays that were examined.

As per the manufacturer's protocol, cDNAs were combined with TaqMan Gene Expression Master Mix (Applied Biosystems) and 100 ng cDNA was added to each well of the custom TaqMan Array Plate and amplified by PCR on an Applied Biosystems 7900HT Fast Real-Time PCR System under the recommended cycling conditions: 2 min at 50C, 10 min at 95C and 40 cycles of 15 sec at 95C for denaturation and 1 min at 60C for annealing/extension. Raw threshold cycle (C_t_) values were obtained using Sequence Detection System (SDS) software v2.4 (Applied Biosystems). C_t_ values ≥40 were set to 40 and were considered not detectable. Among 4 reference genes tested, beta-2 microglobulin (B2M), 18S ribosomal RNA, glyceraldehyde-3-phosphate dehydrogenase (GAPDH), and ubiquitin C (UBC), 18S was found to be the most stable according to analysis with DataAssist Software, v2.0 (Applied Biosystems) and therefore was chosen as the reference gene for normalization of all gene expression results.

For comparative statistics, mRNA data files were processed with Partek Genomics Suite version 6.11.1115 (http://www.partek.com) using default parameters and 18S as the endogenous control. Mean normalized C_t_ values for each assay over all biological replicates (*n* = 2) for both of the experimental comparison groups (HEK293-PRL-1 and HEK293-vector) were calculated. Differential expression between the two comparison groups was determined for each assay using the comparative C_t_ method (ΔΔC_t_) and assessed for significance in terms of p*-*value by the Student's t*-*test. Multiple-testing false discovery rate (FDR) correction values were calculated using the Benjamini-Hochberg procedure [34].

### Data Availability

The microarray and qRT-PCR data sets are available in the NCBI Gene Expression Omnibus (http://www.ncbi.nlm.nih.gov/geo) as series accession number GSE42588.

### Functional, Network, and Pathway Analysis

Two input data sets for functional and pathway analysis of the protein mass-spectrometry results were prepared by applying significance cutoffs of q≤0.20 and q≤0.50 to the detected Tier-1 differentially-expressed proteins. These data sets, consisting of 81 and 172 proteins respectively, included each protein's Entrez Gene ID, fold change under the experimental conditions described in the mass-spectrometry section above, and the p-value and FDR-corrected q-value of that change.

Two input data sets for functional and pathway analysis of the mRNA microarray results were prepared by applying significance cutoffs of q≤0.20 and q≤0.50 to the detected differentially-expressed probesets. These data sets, consisting of 58 and 2438 probesets respectively, included each probeset's Affymetrix ID, associated gene Entrez ID, fold change under the experimental conditions described in the mass-spectrometry section above, and the p-value and FDR-corrected q-value of that change.

For each of the four above input data sets, enriched biological functions and pathways were determined using the DAVID Functional Annotation and Gene Function Classification tools version 6.7 at http://david.abcc.ncifcrf.gov/ [35], [36].

## Results

To investigate the signaling pathways through which PRL-1 mediates its biological effects, we previously established and characterized a HEK293 cell line stably overexpressing PRL-1 and confirmed that both the mRNA and protein levels of PRL-1 in this line are at least 2-fold higher than that of endogenous PRL-1 in the associated vector control cells [11], [16]. The stable overexpression of PRL-1 in the HEK293 cells produces significant changes in the patterns of expression of mRNA transcripts and proteins. In the first part of this section, we examine these changes at the level of the individual nucleic acid and protein experiments. In the second part, we examine these changes using data sets constructed from the integration of the results of nucleic acid and protein experiments.

### Mass Spectrometry

To identify proteins whose expression is specifically altered in response to PRL-1, protein lysates from seven independent cultures each of HEK293-PRL-1 and HEK293-vector cells were subjected to MS analysis. Proteomic analysis resulted in the identification, coding gene annotation, and relative quantification of 763 Tier 1 (high peptide ID confidence; multiple hits) and 571 Tier 2 (high peptide ID confidence; single hit) proteins. Of these, there were 45 Tier 1 and 15 Tier 2 proteins that were subtly, but significantly differentially expressed (FDR ≤0.05) between the HEK293-PRL-1 and HEK293- vector cell lines. 23 Tier 1 and 5 Tier 2 proteins were up-regulated in the HEK293-PRL-1 lines and 22 Tier 1 and 10 Tier 2 proteins were downregulated in these lines with respect to the vector controls. A list of significantly differentially expressed Tier 1 proteins is provided in Table 1. A complete list of identified Tier 1 and 2 proteins is provided in Table S1.

**Table 1:** Significant (q≤0.05) differentially-expressed Tier-1 proteins from mass-spectrometry analysis of PRL-1-overexpressing HEK293 cells.

Protein ID	Coding Gene Name	Entrez Gene ID	Empty Vector Signal	PRL1-Overex. Signal	Fold Change	p-value	FDR
IPI00302592.2	**FLNA**	2316	14630	18061	1.23	1.28E-15	1.2E-12
IPI00550363.2	**TAGLN2**	8407	28555	25728	1.21	2.77E-11	8.5E-09
IPI00026230.1	**HNRNPH2**	3188	18665	22688	1.14	2.68E-04	9.8E-03
IPI00010204.1	**SRSF3**	6428	36303	24669	1.14	8.64E-04	2.2E-02
IPI00010105.1	**EIF6**	3692	13685	19513	1.13	3.36E-04	1.1E-02
IPI00005978.7	**SRSF2**	6427	27477	25050	1.13	2.73E-03	4.8E-02
IPI00465439.4	**ALDOA**	226	26307	15255	1.12	1.34E-03	2.9E-02
IPI00021700.3	**PCNA**	5111	22571	18349	1.11	8.51E-08	8.7E-06
IPI00029079.5	**GMPS**	8833	15137	27233	1.11	3.06E-05	1.4E-03
IPI00009104.6	**RUVBL2**	10856	19000	20909	1.10	1.17E-05	6.3E-04
IPI00021187.3	**RUVBL1**	8607	17014	38789	1.09	4.68E-05	2.0E-03
28935	**ACLY**	47	18448	16848	1.09	9.83E-04	2.4E-02
IPI00017617.1	**DDX5**	1655	24037	25907	1.08	2.88E-04	1.0E-02
IPI00011134.1	**HSPA6**	3310	48728	19488	1.08	8.22E-04	2.2E-02
IPI00012007.5	**AHCY**	191	19038	20153	1.08	1.32E-03	2.9E-02
IPI00014424.1	**EEF1A2**	1917	31227	21229	1.08	2.53E-03	4.6E-02
IPI00645907.2	**FASN**	2194	25369	26014	1.07	3.15E-07	2.6E-05
IPI00301936.3	**ELAVL1**	1994	18210	30686	1.07	1.76E-04	6.7E-03
IPI00304925.3	**HSPA1A**	3303	35223	20761	1.07	2.16E-03	4.1E-02
IPI00027442.4	**AARS**	16	13358	12750	1.06	4.79E-04	1.4E-02
IPI00186290.5	**EEF2**	1938	35443	14098	1.06	6.80E-04	1.8E-02
IPI00013808.1	**ACTN4**	81	20245	41484	1.05	1.05E-03	2.5E-02
IPI00013508.5	**ACTN1**	87	17101	21325	1.04	1.61E-03	3.4E-02
IPI00003881.5	**HNRNPF**	3185	25884	24858	−1.04	1.09E-03	2.6E-02
IPI00645078.1	**UBA1**	7317	21758	29386	−1.05	3.54E-04	1.2E-02
IPI00024067.3	**CLTC**	1213	15241	20481	−1.05	1.28E-03	2.9E-02
IPI00166768.2	**TUBA1C**	84790	53528	50481	−1.06	1.88E-03	3.8E-02
IPI00220644.7	**PKM**	5315	32867	37698	−1.07	3.80E-04	1.2E-02
IPI00643041.2	**RAN**	5901	58484	11372	−1.07	2.53E-03	4.6E-02
IPI00479997.3	**STMN1**	3925	24430	18787	−1.09	4.41E-04	1.3E-02
IPI00329801.11	**ANXA5**	308	16450	30922	−1.09	1.41E-03	3.0E-02
438069	**PRDX2**	7001	21747	42207	−1.09	2.57E-03	4.6E-02
IPI00015018.1	**PPA1**	5464	28822	18988	−1.11	8.25E-05	3.4E-03
IPI00643920.2	**TKT**	7086	25307	9141	−1.12	8.51E-09	1.1E-06
IPI00008557.3	**IGF2BP1**	10642	20573	29331	−1.12	3.51E-06	2.1E-04
IPI00015947.4	**DNAJB1**	3337	22598	41893	−1.12	1.24E-04	4.9E-03
IPI00031461.1	**GDI2**	2665	21957	36925	−1.13	7.64E-08	8.7E-06
5822569	**GSTP1**	2950	17205	27309	−1.13	1.66E-07	1.5E-05
IPI00012048.1	**NME1**	4830	43955	35961	−1.13	1.94E-05	9.9E-04
IPI00291510.3	**IMPDH2**	3615	18005	14479	−1.13	3.03E-05	1.4E-03
IPI00019376.5	**SEPT11**	55752	12830	10236	−1.13	2.05E-03	4.0E-02
IPI00218667.2	**STMN2**	11075	14649	31947	−1.15	6.73E-04	1.8E-02
IPI00217143.2	**SDHA**	6389	11687	19318	−1.15	1.75E-03	3.6E-02
IPI00003815.2	**ARHGDIA**	396	30071	28853	−1.17	6.79E-12	3.1E-09
IPI00418471.5	**VIM**	7431	28883	24494	−1.17	9.93E-11	2.3E-08

Abbreviations: ID  =  identification; Overex.  =  overexpressing; FDR  =  false discovery rate.

### Microarray

Expression changes at the mRNA level were simultaneously analyzed using Affymetrix Human Genome U133 Plus 2.0 microarrays on HEK293 cells that were cultured independently from those utilized in the proteomic analysis. Of the 15,967 microarray probesets that were assayed for mRNA expression and found to be present in one or both comparison groups of HEK293 cells, 25 were found to show significant (q≤0.10) differential expression between PRL-1 overexpressing and vector control groups after adjustment for FDR. Of these probesets, 11 showed decreased levels and 14 showed increased levels following overexpression of PRL-1. Table 2 lists these significantly changing probesets along with their corresponding genes, while Table S2 displays the results for all 15,967 probesets.

**Table 2:** Significant (q≤0.10) differentially expressing mRNA signals from microarray analysis of PRL-1 overexpressing HEK293 cells.

Probeset ID	Gene Symbol	Entrez Gene ID	Empty Vector Signal	PRL1-Overex. Signal	Fold Change	p-value	FDR
200665_s_at	**SPARC**	6678	32	632	20	5.86E-05	0.068
210715_s_at	**SPINT2**	10653	486	2389	4.9	2.46E-05	0.068
213746_s_at	**FLNA**	2316	1080	2402	2.2	6.44E-05	0.068
200859_x_at	**FLNA**	2316	1750	3459	2.0	4.05E-05	0.068
201132_at	**HNRPH2**	3188	2008	3824	1.9	1.04E-04	0.079
203689_s_at	**FMR1**	2332	1961	3603	1.8	1.19E-04	0.083
219569_s_at	**SLC35G2**	80723	868	1556	1.8	4.07E-05	0.068
206546_at	**SYCP2**	10388	68	114	1.7	4.12E-05	0.068
232289_at	**KCNJ12**	3768	192	309	1.6	5.39E-05	0.068
225673_at	**MYADM**	91663	858	1203	1.4	9.79E-05	0.079
1553122_s_at	**RBAK**	57786	177	232	1.3	4.65E-05	0.068
215646_s_at	**VCAN**	1462	1538	2007	1.3	9.03E-05	0.079
219326_s_at	**B3GNT2**	10678	365	455	1.2	1.13E–04	0.082
200874_s_at	**NOP56**	10528	1352	1617	1.2	1.38E-05	0.068
223125_s_at	**C1orf21**	81563	1186	1059	−1.1	4.20E-05	0.068
221194_s_at	**RNFT1**	51136	636	503	−1.3	1.37E-04	0.091
221819_at	**RAB35**	11021	1098	834	−1.3	9.64E-05	0.079
211658_at	**PRDX2**	7001	5648	4248	−1.3	1.45E-04	0.092
217780_at	**WDR830S**	51398	4568	3218	−1.4	3.78E-05	0.068
227590_at	**C22orf40**	150383	566	375	−1.5	6.00E-05	0.068
219029_at	**C5orf28**	64417	750	418	−1.8	6.86E-05	0.068
210414_at	**FLRT1**	23769	232	101	−2.3	3.65E-05	0.068
208966_x_at	**IFI16**	3428	1317	394	−3.3	6.85E-05	0.068
239352_at	**SLC6A15**	55117	447	38	−12	6.28E-05	0.068
225864_at	**FAM84B**	157638	3361	11	−300	1.01E-04	0.079

Abbreviations: ID  =  identification; Overex.  =  overexpressing; FDR  =  false discovery rate.

### Quantitative RT-PCR Validation

The protein coded by the top up-regulated transcript by microarray, SPARC (secreted protein, acidic, cysteine-rich), was not detected in the proteomic data. Therefore, to further validate the microarray result for this gene, SPARC expression was evaluated by quantitative RT-PCR, using two HEK293-PRL-1 and two HEK293-empty-vector samples that were independent from those used for the microarray analysis. As shown in Table 3, qRT-PCR validation confirmed that SPARC mRNA is significantly (q-value  = 0.012, fold-change  = 230) up-regulated in response to PRL-1 overexpression.

**Table 3:** Significant (q≤0.05) differentially expressing mRNA transcripts from qRT-PCR analysis of PRL-1 overexpressing HEK293 cells.

Assay ID	Gene Symbol	Entrez Gene ID	Empty Vector ΔC_t_	PRL-1 Overex. ΔC_t_	Fold Change (2^−ΔΔCt^)	p-value	FDR
Hs00234160_m1	**SPARC**	6678	22.9	15.1	230	1.1E-04	1.3E-02
Hs00181051_m1	**APC**	324	20.6	15.9	25	2.1E-03	3.3E-02
Hs00153074_m1	**ROCK2**	9475	19.6	14.9	25	6.0E-04	2.7E-02
Hs00180679_m1	**PIK3CA**	5290	23.7	19.3	21	1.1E-02	4.9E-02
Hs00232783_m1	**ZEB1**	6935	21.3	17.0	20	4.1E-03	4.3E-02
Hs00179099_m1	**MAP3K2**	10746	20.0	15.8	19	6.7E-03	4.8E-02
Hs00936371_m1	**HIF1A**	3091	19.2	15.1	17	5.3E-03	4.3E-02
Hs00362308_m1	**SOS1**	6654	20.7	16.6	17	5.2E-03	4.3E-02
Hs00182099_m1	**PPP1R12A**	4659	19.9	15.9	16	1.0E-02	4.9E-02
Hs00855199_g1	**ACTR2**	10097	19.4	15.5	14	8.7E-03	4.9E-02
Hs01110394_m1	**ITGB8**	3696	24.7	21.0	14	4.5E-03	4.3E-02
Hs00180035_m1	**NRAS**	4893	17.9	14.1	13	9.3E-03	4.9E-02
Hs01127699_m1	**ROCK1**	6093	19.5	15.8	13	1.1E-02	4.9E-02
Hs00828586_m1	**ACTR3**	10096	17.4	13.8	13	6.6E-03	4.8E-02
Hs01039896_m1	**MAP3K5**	4217	22.1	18.6	11	1.9E-03	3.3E-02
Hs00381459_m1	**PIK3R1**	5295	20.8	17.3	11	7.3E-03	4.9E-02
Hs00168433_m1	**ITGA4**	3676	19.1	15.7	11	3.6E-03	4.3E-02
Hs00601957_m1	**CSNK2A1**	1457	20.0	16.7	10	1.2E-02	4.9E-02
Hs00177373_m1	**MAP3K7**	6885	18.0	14.7	9.9	3.8E-03	4.3E-02
Hs00237216_m1	**NCK1**	4690	20.9	17.6	9.4	1.2E-03	3.1E-02
Hs00375042_m1	**SHC3**	53358	23.4	20.2	8.8	8.9E-03	4.9E-02
Hs00187614_m1	**WASL**	8976	19.9	16.8	8.6	1.1E-02	4.9E-02
Hs00180418_m1	**CRK**	1398	18.2	15.1	8.6	6.1E-03	4.7E-02
Hs00394890_m1	**MAP3K1**	4214	20.3	17.3	7.9	1.2E-02	4.9E-02
Hs00427259_m1	**PPP2CA**	5515	16.0	13.0	7.6	1.2E-02	4.9E-02
Hs00387426_m1	**MAP2K4**	6416	19.6	16.9	6.5	4.0E-03	4.3E-02
Hs01124081_m1	**LAMA2**	3908	26.1	23.5	5.8	8.7E-04	2.7E-02
Hs00177102_m1	**MAPK9**	5601	17.2	14.8	5.5	8.4E-03	4.9E-02
Hs00560189_m1	**PPM1E**	22843	18.9	16.7	4.8	1.0E-02	4.9E-02
Hs00177083_m1	**MAPK8**	5599	17.8	15.5	4.8	4.2E-03	4.3E-02
Hs00373461_m1	**MAPK10**	5602	25.0	22.9	4.4	1.2E-02	4.9E-02
Hs00183311_m1	**SOS2**	6655	19.0	16.8	4.4	3.2E-03	4.3E-02
Hs00180269_m1	**BAX**	581	12.8	10.8	4.1	2.1E-03	3.3E-02
Hs00169777_m1	**PECAM1**	5175	26.0	24.5	3.0	7.5E-03	4.9E-02
Hs00237119_m1	**MMP14**	4323	21.2	19.7	2.8	1.2E-02	4.9E-02
Hs00266332_m1	**COL15A1**	1306	25.2	23.9	2.4	1.3E-03	3.1E-02
Hs00610483_m1	**HRAS**	3265	15.8	14.7	2.1	8.5E-04	2.7E-02
Hs01548727_m1	**MMP2**	4313	19.5	19.2	1.2	9.0E-03	4.9E-02
Hs00174575_m1	**CCL5**	6352	24.4	24.6	−1.2	5.2E-03	4.3E-02
Hs00365167_m1	**COL6A2**	1292	14.9	16.1	−2.2	1.1E-02	4.9E-02
Hs00242448_m1	**COL6A1**	1291	14.7	16.1	−2.7	8.4E-03	4.9E-02
Hs00266026_m1	**IGFBP7**	3490	17.1	18.9	−3.4	1.4E-04	1.3E-02
Hs00609088_m1	**COL5A1**	1289	19.5	21.6	−4.2	1.7E-03	3.3E-02
Hs02341135_m1	**PTP4A3**	11156	16.3	19.0	−6.4	5.1E-03	4.3E-02
Hs00174009_m1	**ITGB4**	3691	19.5	22.5	−8.0	5.9E-04	2.7E-02
Hs00230853_m1	**HNF4A**	3172	22.6	25.9	−9.7	1.1E-02	4.9E-02

Abbreviations: ID  =  identification; Overex.  =  overexpressing; FDR  =  false discovery rate.

Previous studies have shown a relationship between PRL-1 and various components of integrin-mediated cell signaling pathways [11], [12], [14], [37]. These integrin-responsive players can promote re-arrangements in the actin cytoskeleton that are central to promotion of cell motility, invasion, and metastasis. Therefore, a total of 184 genes (including SPARC) known to be associated with integrin-mediated signaling pathways or cytoskeletal remodeling were arrayed on Taqman custom 96-well plates and assayed for differential expression in response to PRL-1 up-regulation. Of the 174 qRT-PCR assays that yielded mRNA expression signals, 46 were found to have significant (q≤0.05) differential expression after adjustment for FDR. Most significantly up-regulated genes represented positive regulators of epithelial-mesenchymal transition (EMT), cell proliferation, survival, and migration, for example HIF1A, ZEB1, H-RAS, N-RAS, ROCK 1/2, Arp 2/3 (ACTR2/3), PIK3CA, and PIK3R1. Among the down-regulated genes were HNF4A, a suppressor of EMT and IGFBP7, a stimulator of cell adhesion and inhibitor of cell growth. The PRL family member PRL-3 (PTP4A3) was also among the down-regulated genes, which is surprising given that PRL-3, like PRL-1, is known to enhance cell growth, invasion, and migration [38]. The reasons for this decrease in PRL-3 expression are currently unknown. Table 3 lists all genes that were determined by qRT-PCR to be significantly differentially expressed. The full list of qRT-PCR assays and results can be found in Table S3.

### Microarray and protein data integration

825 of the 918 Tier-1 proteins detected by mass spectrometry were mapped to a least one probe set on the HG-U133 Plus 2.0 array by coding gene name matching. After accounting for multiple protein products associated with the same coding gene, the final count of unique Tier 1 proteins that were mapped to microarray probesets was 763. Although other groups have demonstrated that some microarray probesets can be associated with the specific mRNA transcripts of particular protein isoforms [39], all protein-mRNA mapping in this study was performed at the more conservative level of the coding gene. Of the 1202 probesets mapped to Tier-1 proteins, 1089 (91%) had detectable gene expression as defined by their presence or absence in either comparison group, demonstrating a high level of co-detection.

**Figure 1:**
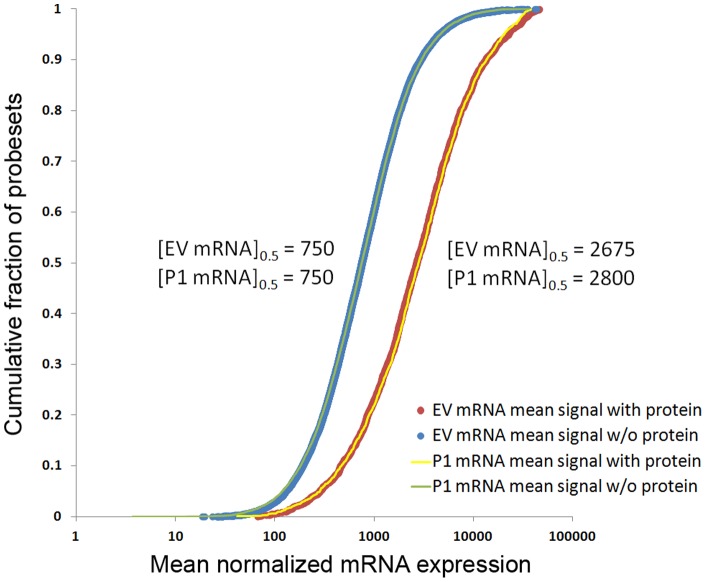
Cumulative distributions of mRNA expression levels for microarray probesets. The cumulative distributions of the expression levels of mRNA probesets that were associated with the coding genes of detected and non-detected proteins are respectively shown in blue and red for the empty-vector (EV) group, and in green and yellow for the PRL-1-overexpressing (P1) group. These data demonstrate that the mean mRNA expression level was approximately 4-fold higher for transcripts corresponding to proteins that were detected (red and yellow) in the proteomic analysis compared to those transcripts where proteins were not detected (blue and green) in the proteomic analysis.

Further evidence of the alignment of the mRNA microarray and protein experimental results is provided by a comparison of the distributions of the expression signals of those mRNA probesets that were matched to coding genes of detected proteins and those that were not. Figure 1 shows that the proteins associated with higher mRNA expression levels were preferentially detected in the mass-spectrometry experiment in both the empty-vector (EV) and the PRL-1-overexpressing (P1) groups compared to proteins whose mRNAs were expressed at lower levels. The medians of the distributions for the protein-matched and non-protein-matched probeset expression values differ by a factor of approximately 4-fold, which is consistent with values reported by other paired protein and mRNA studies [40]. The median expression level for the mRNAs corresponding to proteins that were detected in the PRL-1 transfectants was approximately 5% higher than that observed in the empty vector group.

**Figure 2:**
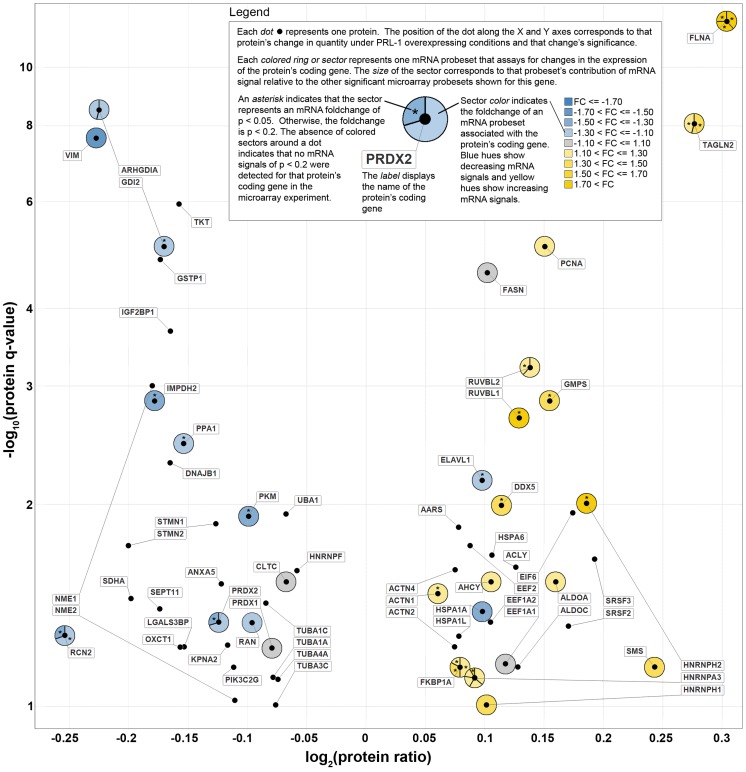
Volcano plot of significant (q≤0.10) differentially-expressed proteins integrated with changes in corresponding mRNA signals . The dot (•) symbols represent the Tier-1 proteins that were observed to be differentially expressed under PRL-1-overexpressing conditions in HEK293 cells. These protein data are plotted along the X- and Y-axes according to the log of the protein expression ratio and FDR-corrected significance respectively. A positive log2(protein ratio) value indicates an up-regulation of protein expression under PRL-1-overexpressing conditions as compared to controls, while a negative value indicates down-regulation of protein expression. Each protein's corresponding mRNA data is represented by a colored circle around that protein's dot symbol. Each probeset in the microarray experiment that was 1) mapped to a plotted protein's coding gene and was 2) differentially expressed with a significance of p≤0.20 is represented by a colored region. An asterisk (*) indicates an mRNA signal with a significance of p≤0.05. In cases where multiple detected probesets were mapped to the same protein's coding gene, the colored circle is divided into sectors according to the relative contribution that each probeset had to the total mRNA signal. Yellow colors represent an up-regulation of mRNA expression and blue colors indicate a down-regulation at the mRNA level. FC  =  fold change; FDR  =  false discovery rate.

We also observed a positive directional correlation between the expression levels of 63 significantly-changed (q≤0.10) proteins and their associated microarray mRNA probesets, as illustrated by the annotated volcano plot in Figure 2. Of the 63 proteins with significant differential expression, 52 were mapped to detected microarray probesets and 30 (48%) had corresponding mRNA level changes at a p-value ≤0.2. There were 43 mRNA transcripts with p≤0.2 that mapped to these 30 proteins. Of these 43 changing transcripts, 39 (91%) demonstrated fold changes in the same direction as the protein and only 4 (mapped to the genes EEF1A1, ELAVL1, FASN, and HSP1A1) changed in the opposite direction.

### Functional and Pathway Analysis

#### Functional annotation enrichment

To address the biological relevance of the significantly differentially regulated proteins and mRNA signals following PRL-1 overexpression, we first used functional annotation enrichment analysis to associate the data with specific biological themes and canonical pathways. The results are summarized below and provided in detail in Dataset S1.

**Figure 3:**
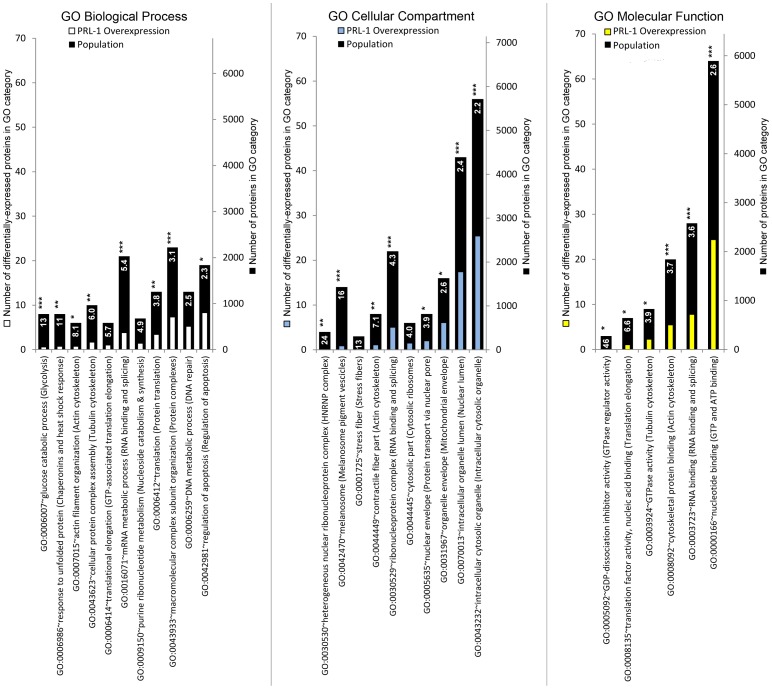
Fold-enrichment of the membership in Gene Ontology categories for differentially expressed Tier-1 proteins under PRL-1 overexpressing conditions in HEK293 cells. The list of 172 Tier-1 proteins that were differentially expressed with a significance of q≤0.5 was submitted to the DAVID server as described in the Methods. For each cluster in the output and for each of the 3 GO classes (Biological Process, Cellular Component, and Molecular Function), the GO term with the highest fold-enrichment and an FDR-corrected significance by Fisher's exact test of q≤0.25 was selected to represent that cluster and represented here as a vertical bar. The author-ascribed description of the cluster itself is appended in parentheses to each GO term bar label on the horizontal axis. The black bar height represents the total population of proteins in a given GO term category. The white, blue, and yellow bar heights represent the number of differentially expressed Tier-1 proteins from the experiment in a given GO term category. The number shown on the bar is the fold-enrichment for a given GO term category, and the stars following each number represent the FDR-corrected significance of that fold-enrichment (no stars for 0.25≥ q>0.1, * for q≤0.1, ** for q≤0.01, *** for q≤0.001). The fold-enrichment number was calculated as (number of Tier 1 proteins in the GO category/number of Tier 1 proteins)/(number of proteins in the GO category/number of proteins in the GO class). GO  =  gene ontology; FDR  =  false discovery rate

Figure 3 shows that the enrichment results from the protein data set indicated an over-representation of coding genes related to high-level (more broad) ontology database annotations of cellular proliferation, tumorigenesis, regulation of cell death, and protein folding (p-value range from 1E-11 to 1E-06). The most enriched low-level (more detailed) annotations were spliceosome components and RNA recognition *via* RNA recognition motif (RRM) domains, nucleotide binding and metabolism (purines in general and GTP in particular), cytoskeletal remodeling (notably actin and intermediate filaments), and integrin-mediated cell-matrix adhesion (p-value range from 1E-12 to 1E-05).

**Figure 4:**
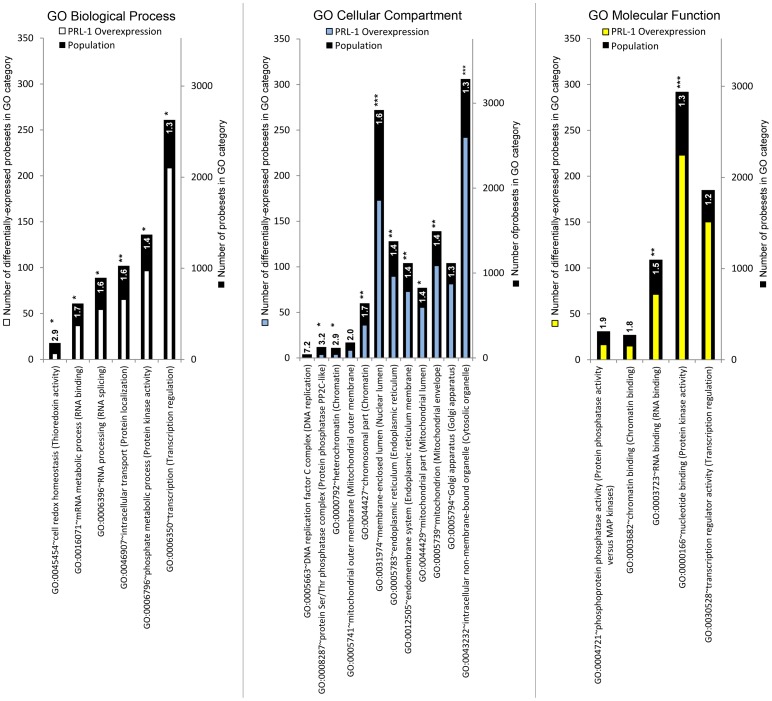
Fold-enrichment of the membership in Gene Ontology categories for differentially expressed mRNA probesets under PRL-1 overexpressing conditions in HEK293 cells . The list of 2438 mRNA probesets that were differentially expressed with a significance of q≤0.5 was submitted to the DAVID server as described in the Methods. For each cluster in the output and for each of the 3 GO classes (Biological Process, Cellular Component, and Molecular Function), the GO term with the highest fold-enrichment and an FDR-corrected significance by Fisher's exact test of q≤0.25 was selected to represent that cluster and represented here as a vertical bar. The author-ascribed description of the cluster itself is appended in parentheses to each GO term bar label on the horizontal axis. The black bar height represents the total population of probesets in a given GO term category. The white, blue, and yellow bar heights represent the number of differentially expressed mRNA probesets from the experiment in a given GO term category. The number shown on the bar is the fold-enrichment for a given GO term category, and the stars following each number represent the FDR-corrected significance of that fold-enrichment (no stars for 0.25≥ q>0.1;* for q≤0.1, ** for q≤0.01, *** for q≤0.001). The fold-enrichment number is calculated as (number of Tier 1 proteins in the GO category/number of Tier 1 proteins)/(number of proteins in the GO category/number of proteins in the GO class). GO  =  gene ontology; FDR  =  false discovery rate.

At the mRNA microarray level, Figure 4 shows that the top functional annotation enrichment results include cellular proliferation, tumorigenesis, RNA recognition and splicing, and cytoskeletal remodeling (p-value range from 1E-07 to 1E-03). The mRNA data also indicate an enrichment in transcriptional regulation terms that is not seen in the protein data, which follows given the greater sensitivity of nucleic acid assays over global protein mass-spectrometry methods when detecting low-abundance regulatory gene products.

#### Pathway analysis

The interactions among the proteins that were differentially-expressed under PRL-1 overexpressing conditions were evaluated in light of previous studies that described PRL-1-associated changes in Rho-mediated signaling pathways [10], [12], [14], the direct interaction between PRL-1 and Rho-regulator ARHGAP4 [19], and the prominence of Rho-regulating proteins in the mass-spectrometry results of this study (e.g. ARHGDIA, GDI2).

**Figure 5:**
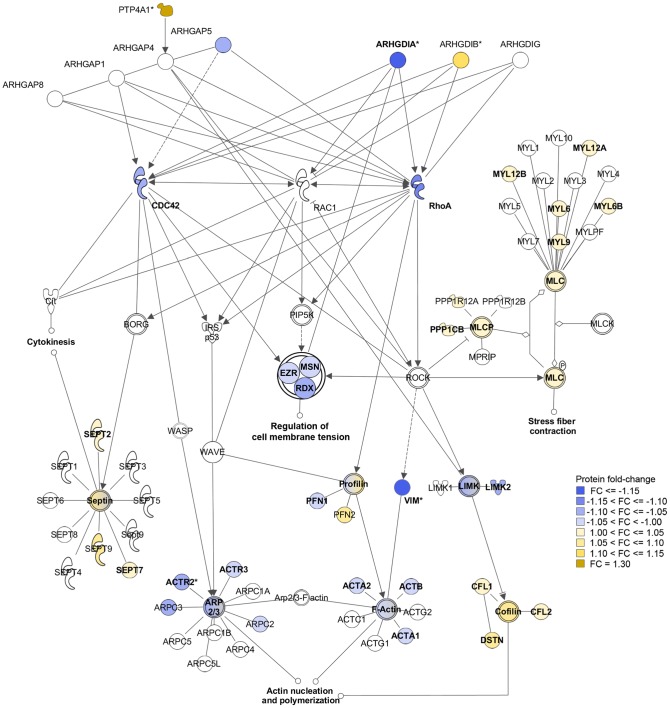
Protein changes in the Rho-signaling canonical pathway resulting from PRL-1 overexpression in HEK293 cells. Selected proteins that conduct signals to remodel the cytoskeleton through RhoA, Rac1, and CDC42 are represented by their coding gene names in a canonical pathway diagram adapted from Ingenuity Pathway Analysis (IPA). The symbols of proteins that were detected in the mass-spectrometry experiment at Tier-1 or Tier-2 levels are colored according to the direction of their fold change (FC) in expression in the PRL-1-transfectants as compared to the empty vector group, with yellow hues indicating an increased quantity of protein and blue hues indicating a decrease. An asterisk (*) indicates that a protein expression change is significant at a level of p≤0.05. Tier-1 proteins are noted with bold font labels. Groups of related or complex-forming proteins are illustrated with double-outlined symbols. Connecting lines with arrowheads indicate an activating, de-activating, or translocating influence, and the absence of an arrowhead indicates a protein-protein binding interaction or group membership. Solid connecting lines show direct interactions while dashed lines show indirect interactions. The known direct interaction of PRL-1 (PTP4A1) with ARHGAP4 is represented here, but indirect connections between PRL-1 and the components of this pathway are not shown.

We observed broad changes in cytoskeletal remodeling signaling proteins in the presence of overexpressed PRL-1. These changes are illustrated in Figure 5 using a diagram of selected direct influences of Rho-regulating proteins on cytoskeleton remodeling that was adapted from the Rho-mediated signaling canonical pathways published in the IPA and GeneGo MetaCore databases. Specifically, we observed a decrease in the expression of Tier-1 Rho guanine nucleotide dissociation inhibitor RhoGDIα (ARHGDIA, foldchange  = −1.17, p = 6.8E-12). RhoGDIα binds to the ezrin-radixin-moesin (ERM) proteins, which regulate membrane-cytoskeletal interactions and maintain membrane tension [41]. All three ERM proteins were detected at Tier-1 and show a non-significant but co-directional decrease in expression [42]. RhoGDIα also binds to RhoA. This interaction not only blocks nucleotide exchange and sequesters RhoA away from its substrates, but additionally protects RhoA from proteosomal degradation [43]. Consequently, RhoA protein expression levels tend to mimic the expression of RhoGDIα [43], [44]. Consistent with this, RhoA protein (but not mRNA) levels were decreased in the PRL-1 transfectants compared to the empty vector controls. This result was further confirmed by western blotting (Protocol S1 and Figure S1). We also observed non-significant, but consistent changes in proteins that drive actin polymerization (*e.g.* actin-related protein 2 or ACTR2 and other members of the ARP2/3 complex), actin disassembly (*e.g.* destrin, cofilin-1, and cofilin-2) and myosin light chain components. The known direct interaction between PRL-1 (PTP4A1) and ARHGAP4 (p115 RhoGAP) [19] is shown in Figure 5, but the previously reported, indirect influences of PRL-1 on the pathway components (*e.g. via* ERK1/2 [11]) are not shown here.

## Discussion

The identification of genes that are affected by PRL-1 up-regulation may provide important clues regarding the biology of this protein and shed light on the mechanism underlying PRL-1 induced tumorigenesis and metastasis. However, there is an expanding repertoire of genes thought to be under PRL-1 control, suggesting that no single, linear signaling pathway can be attributed to its effects. Therefore, we took a systems level approach, using DNA microarray and mass spectrometry technology, to globally characterize the molecular changes in RNA and protein expression that occur, specifically in HEK293 cells stably overexpressing PRL-1. The HEK293 epithelial cell line was chosen to investigate the effects of PRL-1 overexpression because we had previously characterized the phenotypic and functional alterations, including enhanced cell growth and increased migratory and invasive capacity, associated with stable PRL-1 overexpression in this system [11], [16]. Through use of these highly complementary technologies, we have identified several new candidate genes as being responsive to PRL-1 overexpression and provide evidence strengthening the notion that PRL-1 leverages signaling pathways which exert effects mainly on the cell cycle, cytoskeleton, and cellular adhesions to promote cell proliferation and cell survival and to favor the acquisition of invasive and metastatic properties.

### Most genes display coordinate regulation at the mRNA and protein levels

Overall, there was good directional correspondence between the RNA and protein data with 91% of mRNA microarray probesets changing in the same direction as the significantly differentially expressed proteins to which they map. This correspondence implies that the levels of these proteins are driven directly by the abundance of their cognate transcripts. There were a small number of instances where this correspondence did not hold: for example, there were four genes, ELAVL1 (embryonic lethal, abnormal vision, drosophila-like 1), HSPA1A (heat shock 70kDa protein 1A), EEF1A1 (eukaryotic translation elongation factor 1, alpha 1), and FASN (fatty acid synthase) where the protein and RNA showed opposite expression patterns. A lack of correlation between RNA and protein could be due to multiple factors, including differential turnover rates or the presence of post-transcriptional or post-translational control mechanisms. The established thresholds or differential sensitivities and biases between the microarray and proteomics assays could also be a factor. For one, proteomics datasets tend to display a systematic bias favoring more abundant proteins over low abundant, transiently expressed or unstable molecules [45]–[47]. In support of this, an examination of the RNA expression levels revealed that the signal distribution was approximately four times higher for genes whose products were detected in the proteomic survey as compared to those that were not, suggesting that some changes may simply not have been detected due to low protein abundance. Although the mean fold change observed for proteins in this study was much smaller than that observed for mRNA expression, these changes are consistent with mean fold changes in protein expression observed in other cells undergoing EMT [48].

Our combined proteomic and transcriptomic data sets are highly complementary to one another and provide a more complete picture of PRL-1-mediated signaling events, in HEK293 cells, than could be gleaned from either technique in isolation. These data suggest that, in many cases, transcript levels trend the same as protein levels and can be used as a general indicator of protein abundance in this system, but that both transcription-dependent and transcription-independent pathways contribute to PRL-1-induced signaling responses.

### FLNA, HNRNPH2, and PRDX2 are among the most significantly changing gene products in both the microarray and proteomics datasets

A total of 17 genes were identified (those marked with an asterisk in Figure 2) that exhibited statistically significant changes in expression at both the RNA and protein levels and each of these is revealed here, for the first time, to be responsive to alterations in PRL-1 expression. Three of these genes, FLNA, HNRNPH2, and PRDX2 continued to reach significance at both the RNA and protein levels, even after multiple testing correction was applied to both data sets and therefore make highly promising candidates for downstream components of the PRL-1 signaling pathway.

FLNA (Filamin A) represents the most robustly and highly up-regulated protein in the proteomic analysis. In addition, all three probesets for FLNA on the Affymetrix microarray showed approximately 2-fold up-regulation in response to PRL-1 (p<0.05). The FLNA gene encodes the most abundant and widely expressed member of a family of three filamin proteins (FLNa, FLNb, FLNc) [49]. It is a large, homodimeric, actin binding protein that plays important roles in remodeling the cytoskeleton to influence cell shape and cell motility [49]–[51]. Cells deficient in FLNa exhibit defects in both cell spreading and initiation of migration [52].

Filamin A also serves as a versatile molecular scaffold. It directly interacts with more than 90 different proteins including transmembrane receptors, ion channels, intracellular signaling molecules, and transcription factors [53]. Among these are several members of the integrin and Rho GTPase families which play central roles in actin cytoskeletal reorganization, cell adhesion, cell migration, invasion, and control of cell cycle progression [54]–[56]. FLNa can bind the Rho GTPases Rho, Rac, and Cdc42, the Rac guanine nucleotide exchange factor (GEF) Trio, the RhoGEF Lbc, the Rho GTPase activating protein p190RhoGAP, the Rac GAP FilGAP, and the Rho GTPase effectors PAK and ROCK [50]. These interactions make FLNa an ideal integrator of the Rho GTPase signaling cascade. Changes in PRL-1 expression have previously been shown to alter the levels of GTP-bound RhoA, RhoC, Rac1, and Cdc42 [10], [12]. Moreover, PRL-1 overexpression in the current study led to downregulation of RhoGDIα and RhoA expression levels. The strong up-regulation of FLNa in the PRL-1 transfectants, combined with its known relationship to the Rho pathways make it an attractive subject for future examination as a potential link between PRL-1 and control of Rho GTPase-mediated signaling.

HNRNPH2 was also significantly up-regulated at both the mRNA and protein levels in response to PRL-1. This molecule belongs to the heterogenous nuclear ribonucleoprotein (hnRNP) family of RNA-binding proteins which heavily influence pre-mRNA processing as well as other aspects of mRNA metabolism and transport [57], [58]. The hnRNPH2 protein is part of a subfamily of hnRNP whose members (H1, H2, H3, and F) are best known for their key roles in the regulation of alternative splicing. Splice site selection is controlled by the orchestrated effects of multiple splicing factors that bind to specific RNA elements and either promote or impede the assembly of the splicing machinery [59], [60]. In addition to the HNRNP family, other gene families with well known roles in alternative splice site selection include the serine/arginine-rich splicing factor (SRSF) family [60] and the embryonic lethal, abnormal vision, *Drosophila*-like (ELAVL) family [61], [62]. Notably, in our study, members of each of these three families of splice site regulators (HNRNPH1, HNRNPH2, HNRNPF, HNRNPA3, SRSF2, SRSF3, and ELAVL1) exhibited significant changes in expression, at least at the protein level, upon PRL-1 overexpression. Alternative splicing increases the functional complexity of gene expression and, in tumors, it generates variants that can contribute to multiple aspects of tumor establishment, progression, and maintenance. Observations suggest that genes involved in cell morphology, movement, adhesion, growth, proliferation, and cytoskeletal organization are particularly prone to alternative splicing events [63]. Genes involved in each of these processes have been shown, both here and in other studies, to be modulated by PRL-1 raising the possibility that changing alternative splicing patterns may be one mechanism by which PRL-1 contributes to cancer cell plasticity.

In contrast to FLNA and HNRNPH2, the gene products of PRDX2 were significantly down-regulated upon PRL-1 overexpression. PRDX2 is a member of the peroxiredoxin (Prdx) family of ubiquitously expressed antioxidant enzymes with important functions in maintaining cellular redox homeostasis. Studies have shown that inactivation of Prdx family members may be necessary for hydrogen peroxide mediated cellular signaling in response to growth factor stimulation and for cell survival signaling under conditions of oxidative stress [64], [65]. However, elevated levels of each Prdx family member have been found in a variety of cancer cell lines and tissues [66]–[69] and Prdx2 can directly suppress the activity of several pro-apoptotic factors [67]. Therefore, the functional consequences of Prdx activity and/or inhibition remain an active area of study.

Taken together, the consistent and robust changes of RNA and protein for FLNA, HNRNPH2, and PRDX2, provides strong confidence that these alterations can be attributed to PRL-1 overexpression and make these attractive candidates for further investigation.

### The matrix associated gene SPARC (osteonectin) is the most significantly up-regulated gene at the mRNA level

Most PRL-1-induced differences in expression were less than two-fold in magnitude, however, SPARC transcripts were shown by the Affymetrix microarray to be up-regulated 20-fold (p = 5.86E-05) in the PRL-1 transfectants compared to vector control cells. SPARC (also known as osteonectin) is a non-structural, extracellular matrix (matricellular) glycoprotein that is involved in matrix remodeling and influences a diverse array of biological processes [70], [71]–[73]. SPARC influences cell-cell and cell-matrix interactions; promotes extracellular matrix remodeling; regulates integrin expression and activity; alters focal adhesions; and modulates the activity of growth factors, cell cycle regulators, matrix metalloproteinases, and molecules involved in cytoskeletal rearrangement. It thereby controls a wide range of cellular functions, including cell cycle progression, cell proliferation, cell survival, angiogenesis, migration, invasion and metastasis.

Although qRT-PCR validation, in an independent sample set, reproducibly confirmed the significant up-regulation of SPARC message in the HEK293-PRL-1 transfectants, no protein product was detected for this gene in either the PRL-1 overexpressing or the control cell lines. However, SPARC is a secreted protein and internalized SPARC is thought to be quickly re-released outside the cell [74], which could explain our inability to detect SPARC protein in whole cell lysates. Differential RNA and protein stability could also play a role given that SPARC message has been found to be stable for more than 38 hours [75], while SPARC protein has a half-life of less than two hours [76]. Limitations described in the previous section, regarding low abundance proteins, could also be a factor. Nevertheless, overexpression of PRL-1 in HEK293 cells clearly leads to enhanced levels of SPARC mRNA transcripts, which could play a role in mediating the signaling events downstream of PRL-1. Further supporting this notion, qRT-PCR analysis revealed that FAK (PTK2), SHC, and the Ras pathway, all which are known to lie immediately downstream of SPARC, were also up-regulated in response to PRL-1 overexpression.

Several parallels exist between SPARC and PRL-1 signaling. When overexpressed in epithelial cells, both genes induce morphological and biological changes consistent with EMT [11], [12], [77]. Each has pleiotropic functions with the capacity to enhance cellular proliferation and metastatic potential, but also playing important roles in cellular differentiation [1], [78]. Both molecules display similar tumor type specific influences on human tumor tissues [17], [70], [71], [79]–[82]. Both can also exert similar effects on downstream signaling pathways and molecules such as E-Cadherin [12], [83], Src [11], [84], FAK [11], [83], ERK1/2 [11], [85], MMP2 [11], [86], Akt, p53, p21^cip1/waf1^ [23], [87], [88], and the Rho GTPase family members [12], [84]. Moreover, both genes have been implicated in maintenance of retinal function [5], [89], [90]. Both display age-dependent changes in expression with an inverse correlation to age in the skeletal muscle [17], [91] and a positive correlation to age in structures of the brain [17], [92]. And finally, both genes exhibit cell cycle dependent localization of expression [18], [93]. During mitosis, PRL-1 interacts directly with α-tubulin and localizes to the centrosomes, where it has been suggested to play a role in modulating spindle dynamics [18]. Interestingly, the integrin-linked kinase (ILK), which is a SPARC interaction partner and a known effector of SPARC signaling [94], also localizes to the centrosome in mitotic cells, where it binds to the RuvB-like proteins 1 and 2 (RUVBL1, RUVBL2), which were both significantly up-regulated in the HEK293-PRL-1 cells. Together, ILK, RUVBL1, and RUVBL2 regulate microtubule dynamics and mitotic spindle organization [95]. ILK also connects to Filamin A through the filamin-binding protein Migfilin [96]. These many commonalities between the PRL-1 and SPARC signaling pathways, along with the up-regulation of SPARC transcripts in response to PRL-1, make SPARC an attractive candidate for future study as a potential mediator of PRL-1 function.

### Altered levels of gene products involved in cytoskeletal rearrangements are a common theme with PRL-1 overexpression

Dynamic reorganization of the cytoskeleton is the primary mechanism by which cells generate the protrusive structures and contractile forces necessary for cell movement [97]–[100]. Cytoskeletal changes also play a crucial role in the orchestration of cell division [101], [102]. In this study, transcriptomic and proteomic analysis revealed that stable overexpression of PRL-1 significantly alters the RNA and/or protein levels of a number of molecules with roles in the assembly, organization, and regulation of each of the three main structural components of the cytoskeleton. PRL-1 overexpression led to significant up-regulation of actin-binding and cross-linking proteins such as FLNA, transgelin-2 (TAGLN2), and the alpha-actinin isoforms ACTN1, ACTN2, and ACTN4. Conversely, overexpression of PRL-1 caused the significant down-regulation of tubulin isoforms (TUBA1A, TUBA4A, TUBA1C, TUBA3C), the microtubule regulators RAN and stathmin (STMN1 and STMN2), the intermediate filament protein vimentin (VIM), and the regulator of Rho signaling RhoGDIα. These data suggest that PRL-1 can modulate cytoskeletal changes at multiple levels. Moreover, the known interaction between PRL-1 and α-tubulin [18] suggests that the influence of PRL-1 on the various isoforms of α-tubulin may be direct.

It deserves mention that up-regulation of vimentin is one of the hallmarks of conversion from an epithelial to a mesenchymal phenotype and expression of vimentin is typically correlated with enhanced cell migration and invasive activity [103]. Thus, we were surprised to find that both vimentin RNA and protein were slightly down-regulated in the PRL-1 transfectants, especially considering that PRL-1 overexpression visibly alters the morphology of HEK293 cells, causing them to elongate and take on a more fibroblast-like appearance and also results in a gain of invasive motility, both changes that are consistent with EMT [104]. Vimentin expression levels have also previously been reported to positively correlate with the expression of FLNA [105] and SPARC [77], hence the mechanisms leading to down-regulation of vimentin in the present study are currently unclear. In some cell types, down-regulation of vimentin has been proposed to inhibit apoptosis, contributing to cell survival and resistance to various anti-cancer agents [106]–[108]. Therefore it is plausible that PRL-1-mediated down-regulation of vimentin could provide HEK293 cells with a survival advantage.

Further supporting the ability of PRL-1 to exert strong influences on the cytoskeleton, members of the Rho signaling pathway and molecules that feed into this pathway were highly over-represented among both significant and non-significant differentially expressed gene products. Alterations in several molecules downstream of the Rho GTPases that mediate actin polymerization and disassembly are consistent with the occurrence of active cytoskeletal remodeling in these cells. Many other molecules with known or suspected roles in the regulation of cytoskeletal reorganization and cell migration also displayed significantly altered expression in response to PRL-1, including SPARC [84], [109], ELAVL1 [110], HSPA1A (Hsp70) [111], EIF6 [112], EEF1A1 [113], IGF2BP1 [114], NME1 [115], NME2 [116], SEPT11 [117], LGALS3BP [118], SPINT2 [119], VCAN [120], MYADM [121], RAB35 [122], FLRT1 [123], and FAM84B [124]. Accordingly, functional enrichment analysis showed an over-representation of genes related to cytoskeletal remodeling and cell adhesion. Up-regulation of gene products involved in nucleotide, nucleic acid, protein, and lipid biosynthesis was also a common theme, consistent with an increased rate of proliferation in the PRL-1 overexpressing cells.

Taken together, all of the above data support a role for PRL-1 in modulation of cytoskeletal components and cytoskeletal regulators to influence cell proliferation, survival, invasion, and migration. Given that PRL-1 significantly up-regulates Filamin A and down-regulates RhoGDIα and RhoA in this system; that Filamin A is known to control the early phases of cell spreading and migration initiation [52]; and that an initial inhibition of RhoA is necessary early on to allow membrane extension during cell spreading [54]; the current evidence may implicate a role for PRL-1 in the very early stages of cell spreading and migration, at least in HEK293 cells.

## Conclusions

The systems level analyses performed in this study offered the opportunity to gain a more broad picture of signaling downstream of PRL-1 in the HEK293 cell line than ever previously obtained. This approach also allowed us to identify, in an unbiased manner, several candidate genes that may not otherwise have been associated with PRL-1 signaling. In particular, Filamin A, RhoGDIα, and SPARC are attractive subjects of future study given their established relationships with a number of signaling molecules (*e.g.* the Rho GTPase family) known to be influenced by PRL-1 expression. In addition to Filamin A, RhoGDIα, and SPARC, PRL-1 was found to significantly alter the expression of multiple other genes with roles in regulation of cell shape, adhesion, motility, and the cell cycle, supporting prior evidence that PRL-1 may control cytoskeletal dynamics and cell division. In particular, members of the Rho signaling pathway appear to be heavily influenced by PRL-1 overexpression. PRL-1 also has strong influence on the expression of genes involved in alternative splicing, presenting another possible mechanism by which PRL-1 may contribute to the acquisition of a tumorigenic and/or metastatic phenotype. This study represents the first comprehensive overview of the biological impact of PRL-1 overexpression on cellular mRNA or protein levels. It is clear from these results that the effects of PRL-1 are much broader than we currently understand. Although further studies will be required to characterize and examine the consequences of the interactions between PRL-1 and the PRL-1 responsive molecules identified here, these results provide a rich resource of information that should serve as a starting point to open up new lines of investigation into the role of this important oncogene.

## Supporting Information

Figure S1 **RhoA protein expression.** Western blot showing RhoA protein levels in HEK293 cells that were transfected with either PRL-1 or with empty vector. GAPDH was used as a loading control.
(DOCX)

Table S1 **Protein mass spectrometry data.** List of Tier 1 and Tier 2 proteins detected from mass spectrometry analysis of HEK293 cells transfected with PRL-1 (P1) or with empty vector (EV).
(XLSX)

Table S2 **mRNA microarray data.** Full list of microarray probesets that were analyzed for differential expression between HEK293 cells transfected with PRL-1 or with empty vector and that passed the presence filter described in the methods.
(XLSX)

Table S3 **Quantitative RT-PCR data.** Full list of genes examined using qRT-PCR analysis of HEK293 cells that were transfected with PRL-1 or with empty vector. A value of not applicable (N/A) indicates that the gene was undetectable (Ct ≥40) in all assayed samples.
(XLSX)

Dataset S1 **DAVID analysis.** Full results from DAVID gene set enrichment analysis of mRNA transcripts and proteins that were differentially expressed in HEK293 cells overexpressing PRL-1, as compared to those transfected with empty vector.
(XLSX)

Protocol S1 **Western blot protocol**.
(DOCX)
