# Supplementary material for: Integrated Analysis of Global mRNA and Protein Expression Data in HEK293 Cells Overexpressing PRL-1
Source: PLoS One. 2013 Sep 3;8(9):e72977. doi: 10.1371/journal.pone.0072977 (PMC3760866; doi:10.1371/journal.pone.0072977)
Supplement: Figure S1 — RhoA protein expression. Western blot showing RhoA protein levels in HEK293 cells that were transfected with either PRL-1 or with empty vector. GAPDH was used as a loading control. (DOCX) [file pone.0072977.s001.docx]

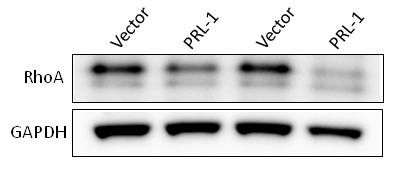


**Figure S1.** Western blot showing RhoA protein levels in HEK293 cells transfected with PRL-1 or with empty vector. GAPDH was used as a loading control.
