# Supplementary material for: Integrated Analysis of Global mRNA and Protein Expression Data in HEK293 Cells Overexpressing PRL-1
Source: PLoS One. 2013 Sep 3;8(9):e72977. doi: 10.1371/journal.pone.0072977 (PMC3760866; doi:10.1371/journal.pone.0072977)
Supplement: Protocol S1 — Western blot protocol. (DOCX) [file pone.0072977.s006.docx]

# Dumaual and Steere, Supplemental Protocol S1

## Western blot analysis of RhoA

HEK293-PRL-1 and HEK293-vector cells were grown to 80% confluency then rinsed with 1X PBS and lysed in RIPA Buffer (Thermo Scientific, Rockford, IL) plus 1X Halt Protease and Phosphatase Inhibitor Cocktail (Thermo Scientific). Cell lysates were sonicated for 5min at 4 C in a Branson 2510 sonicator (Thomas Scientific, Swedesboro, NJ), then centrifuged at 21,000 x g for 20 min at 4 C. Supernatant protein concentrations were determined using the Pierce BCA Protein Assay Kit (Thermo Scientific) and a SpectraMax Plus Microplate Spectrophotometer (Molecular Devices, Sunnyvale, CA), both according to the manufacturer’s protocols. Samples were mixed 1:1 with 6X Laemmli reducing sample buffer (Boston Bioproducts, Ashland, MA) and 15μg of each sample was resolved using NuPAGE 4-12% Bis-Tris gels and NuPAGE 1X MOPS Running Buffer (Invitrogen Life Technologies). Samples were electro-transferred to nitrocellulose membranes using the iBlot Western Blotting System (Invitrogen Life Technologies) and membranes were blocked for 1 hr in 5% BSA in 1X TBST. Blots were incubated in primary RhoA antibody (1:1000; Cell Signaling Technology, Danvers, MA) at 4 C overnight, washed 4X, 2-3 min in TBST, incubated in secondary antibody (1:1000; HRP-linked anti-rabbit-IgG; Cell Signaling Technology) for 1 hr at room temperature, and rinsed 4X, 2-3 min each in TBST again. Membranes were developed using the Pierce SuperSignal West Pico Chemiluminescent Substrate (Thermo Scientific). Images were captured using an ImageQuant LAS-4000 imager (GE Healthcare, Uppsala, Sweden). GAPDH (Cell Signaling Technology) was used as a loading control.
